# Charting the immune terrain: a novel risk model for thyroid cancer prognosis

**DOI:** 10.3389/fgene.2026.1752017

**Published:** 2026-04-23

**Authors:** Qi Qi, Xiaoyan Cai, Qiang Lv

**Affiliations:** Department of General Surgery, Shanghia Pudong New Area Gongli Hospital, Shanghai, China

**Keywords:** immune genes, immune infiltration, prediction model, prognostic risk, thyroid cancer

## Abstract

**Objectives:**

To construct a prognostic risk model for thyroid cancer based on immune genes and analyze the correlation between immune genes and immune infiltration.

**Methods:**

A retrospective study was conducted on 180 patients with thyroid cancer treated in our hospital during May 2022 to April 2025. Based on the prognosis, the subjects were graded as good prognosis group of 126 cases and poor prognosis group of 54 cases. The influencing factors were analyzed by a binary logistic regression model, receiver operating characteristic curve and goodness of fit test. Single sample gene set enrichment analysis was used to perform immune infiltration analysis on the expression matrix of peripheral blood mononuclear cells. The GSEA algorithm was used to calculate the abundance of tumor associated immune cell infiltration. Pearson correlation analysis was used to investigate the correlation. The TCGA-THCA database was used to analyze the differential expression of genes, as well as the correlation with clinical pathological features.

**Results:**

The expression levels of CDK1, B3GNT7, S100A9, and MMP9 genes were higher in the poor prognosis group than the good prognosis group (*P* < 0.05). A prognostic prediction model was constructed according to formula [1/1 + exp (4.125 + 1.250 × CDK1 + 1.880 × B3GNT7 + 0.920 × S100A9 + 1.050 × MMP9)]. The average C-index of the model was 0.919 (95% CI: 0.882–0.961). The AUC of the prognosis prediction model was 0.880. The poor prognosis group had much lower infiltration abundance of B lymphocytes, CD4^+^T lymphocytes, and CD8^+^T lymphocytes, and higher infiltration abundance of neutrophils and macrophages than the good prognosis group (*P* < 0.05). CDK1, B3GNT7, S100A9, and MMP9 were negatively correlated with the infiltration abundance of B lymphocytes, CD4^+^T lymphocytes, and CD8^+^T lymphocytes, and positively correlated with the infiltration abundance of neutrophils and macrophages (*P* < 0.05). Further analysis from the TCGA-THCA database showed that the high expression of S100A9 and MMP9 was correlated with advanced lymph node metastasis (pN stage), distant metastasis (pM stage) and overall TNM stage (*P < 0.05*).

**Conclusion:**

CDK1, B3GNT7, S100A9, and MMP9 were independent risk factors for poor prognosis in thyroid cancer. The prognostic prediction model may provide objective evidence for early screening of high-risk cases in clinical practice.

## 1 Introduction

Thyroid cancer is one of the fastest growing malignant tumors worldwide in incidence rate, of which papillary thyroid cancer accounts for 80%–90% of all thyroid cancers (Boucai et al., 2024). According to data from the International Agency for Research on Cancer, there were as many as 586,000 new cases of thyroid cancer worldwide in 2020, with female patients accounting for three times the proportion of male patients. Early screening of tumors and targeted treatment can effectively improve patient survival rates (Pizzato et al., 2022). In recent years, the dynamic infiltration of immune cells in the tumor microenvironment (TME) and their interaction with genomic features have gradually become key biological indicators for prognosis (Xu et al., 2022).

Basic research reveals that cyclin dependent protein kinase 1 (CDK1) can play a key role in regulating the cell cycle of thyroid cancer cells by catalyzing F-box protein 28 to activate E3 ubiquitin ligase complex. In addition, high expression of CDK1 is associated with tumor cell immune escape (Xiang et al., 2022). Recent studies have shown that β-1,3-N-acetylglucosamine transferase 7 (B3GNT7), as a key glycosyltransferase in tumor immune response, mediates sugar chain modifications that can regulate the expression of immune checkpoint molecules on the surface of thyroid cancer cells, thereby affecting T cell infiltration (Nag et al., 2018). Another study found that S100 calcium binding protein A9 (S100A9) activated calcium channel receptors on the membrane of thyroid cancer cells, promoted the secretion of chemokines, mediated neutrophil infiltration, and formed an immunosuppressive microenvironment (Ito et al., 2009). The latest research confirms that matrix metalloproteinase 9 (MMP9) can degrade the basement membrane barrier of thyroid cancer and release angiogenic factors. The expression level of MMP9 is significantly correlated with the polarization state of macrophages in the tumor (Toraih et al., 2023). However, existing research mostly focuses on the analysis of single immune markers or cell subpopulations, lacking systematic integration of immune gene expression profiles and immune infiltration heterogeneity, making it difficult to establish accurate prognostic evaluation models. Therefore, exploring the expression characteristics of immune cell genes and the regulatory mechanisms of their infiltration levels in the tumor microenvironment, and constructing a prognostic risk model based on immune genes, is of great significance for individualized prognostic evaluation and immunotherapy target screening of thyroid cancer.

In this study, a prognostic model was constructed based on immune genes including CDK1, B3GNT7, S100A9, and MMP9. The correlation with immune cell infiltration was explored, aiming to provide a basis for immunotherapy research of thyroid cancer.

## 2 Materials and methods

### 2.1 General materials

A retrospective study was conducted on 180 patients with thyroid cancer treated in our hospital during May 2022 to April 2025. Based on the prognosis, the subjects were graded as the good prognosis group of 126 cases and a poor prognosis group of 54 cases. This study was ratified by the Ethics Committee of our hospital.

### 2.2 Inclusion and exclusion criteria

**FIGURE 1:**
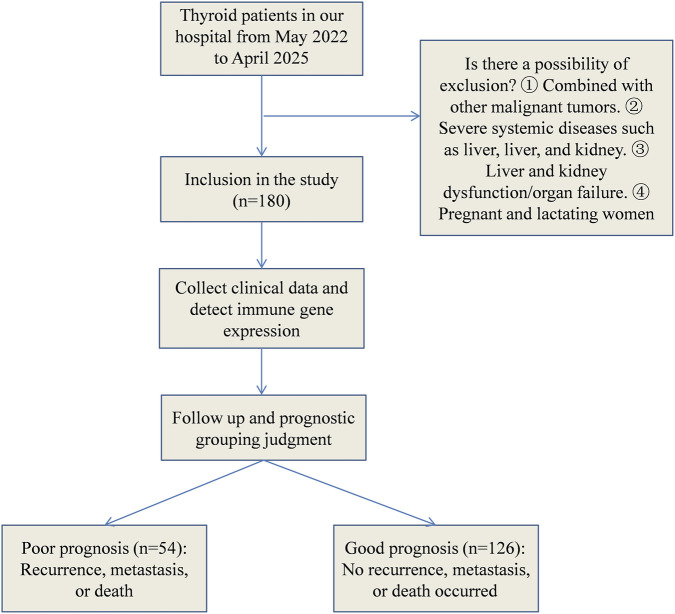
Diagram of data screening process.

Inclusion criteria: (1) All patients met the diagnostic criteria for thyroid cancer (Alonso-Gordoa et al., 2024), and the tissue specimen was confirmed by pathology. (2) All patients underwent radical thyroidectomy for treatment. (3) The clinical data and postoperative pathological reports of thyroid cancer were complete. Exclusion criteria: (1) Patients combined with other malignant tumors; (2) Patients combined with serious systemic diseases such as heart, liver, brain, kidney, etc.,; (3) Patients combined with liver and kidney dysfunction or other organ failure; (4) Pregnant and lactating women. The selection process for the general data was shown in Figure 1.

### 2.3 Grouping criteria

Criteria for recurrence assessment: (1) During postoperative follow-up, symptoms such as neck masses, hoarseness, and dysphagia occurred. (2) Imaging examinations (thyroid ultrasound, CT) showed new occupying lesions in the thyroid bed or cervical lymph nodes, with unclear boundaries, irregular shapes, and accompanied by microcalcification. (3) Recurrent thyroid cancer was confirmed by fine-needle aspiration biopsy pathology.

Criteria for distant metastasis determination: (1) Abnormal radioactive concentration foci were detected in a whole-body bone scan, which are confirmed as bone metastases by CT or MRI. (2) New pulmonary nodules were found in chest CT, and they were confirmed as metastatic foci by pathology or follow-up imaging. (3) Liver metastases were detected by abdominal ultrasound or CT. (4) Distant metastases in other sites (such as brain, adrenal glands, *etc.*) were confirmed by imaging and/or pathology.

Death determination criteria: Death of patients was confirmed through outpatient and inpatient medical record systems, as well as through telephone follow-ups. The time of death and the cause of death (related to thyroid cancer or other causes) were recorded.

Prognostic grouping criteria: In this study, “poor prognosis” was defined as recurrence, distant metastasis, or thyroid cancer-related death during the follow-up period. The reason for using a composite outcome as the research endpoint was as follows: (1) The overall prognosis of thyroid cancer was good, with a 5-year survival rate exceeding 95%. If only death was used as the endpoint, the event rate was low, making it difficult to conduct effective statistical analysis. (2) Recurrence and distant metastasis were key nodes in the progression of thyroid cancer, closely related to the subsequent quality of life and treatment strategies of patients, and have clear clinical significance. (3) Previous studies on the prognosis of thyroid cancer have widely used disease-free survival (DFS) or recurrence-free survival (RFS) as the research endpoint. Incorporating recurrence and metastasis into the composite outcome was in line with the research conventions in the field.

### 2.4 Collection of clinical data

The clinical data of the included subjects were collected, including the age (≤60 years, >60 years), tumor size (≤2cm, >2 cm), tumor type (follicular, papillary, mixed), lymph node metastasis (yes, no) and capsule invasion (yes, no).

### 2.5 Methods

#### 2.5.1 Immune related gene screening strategy

**FIGURE 2:**
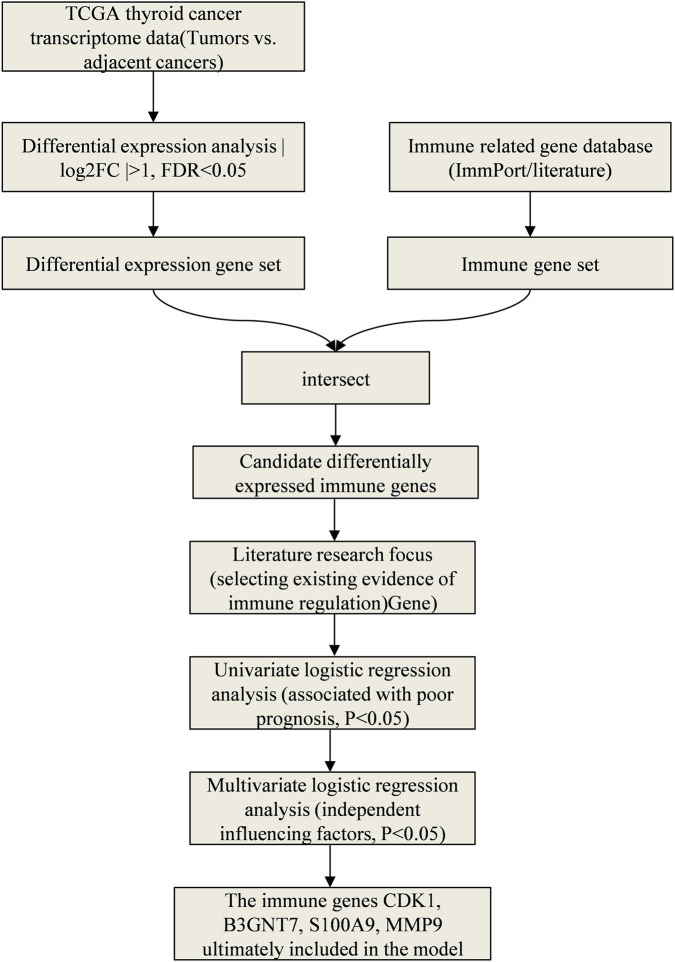
Flowchart for screening immune-related genes.

This study screened the immune genes related to the prognosis of thyroid cancer through the following steps: (1) Downloaded the transcriptome data and clinical information of thyroid cancer from the TCGA database, and used the “limma” package to screen the immune-related genes with differential expression between tumors and adjacent tissues (|log_2_FC| > 1, FDR <0.05). (2) Combined with literature research, focused on the genes that had been confirmed in previous studies to be involved in tumor immune regulation. (3) Conducted a preliminary assessment of the association between each gene and the patient’s prognosis using univariate Logistic regression analysis, retaining the genes with *P* < 0.05. (4) Further screened independent influencing factors through multivariate Logistic regression analysis, and finally determined CDK1, B3GNT7, S100A9, and MMP9 to be included in the model. This strategy referred to the multi-step feature selection methods in recent studies (Mallardo et al., 2024; Mallardo et al., 2023), ensuring the transparency and reproducibility of the screening process. The specific screening process was shown in Figure 2.

#### 2.5.2 Gene expression detection and immune infiltration analysis

Real time fluorescence quantitative PCR (qRT-PCR) was used to detect the mRNA expression levels of CDK1, B3GNT7, S100A9, and MMP9 in tumor tissues. The specific steps were as follows: 180 thyroid cancer tissue specimens were obtained from patients who underwent radical thyroidectomy in our hospital during May 2022 to April 2025. All specimens were diagnosed with thyroid cancer by postoperative pathology and immediately stored in a −80 °C freezer after surgical resection. Total RNA was extracted from tissues using the TRIzol method. After the purity was detected by NanoDrop, cDNA was synthesized using a reverse transcription kit (TaKaRa). Using β-actin as an internal reference, amplification was performed using SYBR Green PCR kit (TaKaRa), and the primer sequences were shown in Table 1. The reaction conditions were as follows: pre denaturation at 95 °C for 30 s, denaturation at 95 °C for 5 s, annealing at 60 °C for 30 s, a total of 40 cycles. Relative gene expression level was calculated using the 2^−△△^Ct method. This study was approved by the hospital Ethics Committee to comply with the Helsinki Declaration and the Clinical Research Ethics Review Measures. Clinical data was anonymized and stored in an encrypted database to ensure privacy protection and data security. The primer sequences were shown in Table 1.

**TABLE 1:** Primer sequences of core immune genes.

Genes	Upstream primer (5’→3′)	Downstream primer (5’→3′)
CDK1	TAG​CAT​CAT​CAG​ATG​CAC​GC	GTG​TCC​AGC​ACT​CGC​ACT​AT
B3GNT7	ACC​GAG​TCT​ATA​TCC​CCT​CTG​A	GCC​ATC​CAC​TGG​CTG​CTA​AC
S100A9	CCA​CAG​CCA​AGA​CAG​TTT​GA	CAG​CTG​GAA​CGC​AAC​ATG​AGA
MMP12	AGT​TAC​CTT​CAA​AGG​CCA​AGA​GA	AGT​CCA​AGG​ATG​TTA​GGA​ACG​A

Detection of immune cell infiltration abundance in tumor tissues: This study employed immunohistochemical staining combined with flow cytometry to quantitatively assess the abundance of immune cells infiltrating tumor tissues. The immunohistochemical staining process included tissue fixation, dehydration and embedding, slice preparation, antigen repair, and incubation with primary antibodies. The primary antibodies used were anti-CD20 antibody for B lymphocytes, anti-CD4 antibody for CD4^+^ T lymphocytes, anti-CD8 antibody for CD8^+^ T lymphocytes, anti-CD15 antibody for neutrophils, and anti-CD68 antibody for macrophages. After staining, the tissues were digested into single-cell suspensions, labeled with corresponding fluorescent secondary antibodies, and then analyzed using a flow cytometer. The FlowJo software was used to analyze the proportion of various immune cell subset in the total cell and expressed the infiltration abundance in percentage form. This method was used to directly quantify the true infiltration level of immune cells in tumor tissues of patients in our hospital cohort.

Bioinformatics immune infiltration analysis: Single-sample gene set enrichment analysis was used to evaluate the association between immune gene expression and immune infiltration. Gene expression profile data of thyroid cancer were downloaded from the TCGA database, and the ssGSEA algorithm was run using the “GSVA” R package to calculate the enrichment scores of B lymphocytes, CD4^+^ T lymphocytes, CD8^+^ T lymphocytes, neutrophils, and macrophage characteristic gene sets in each sample, reflecting the relative infiltration abundance of each immune cell type. This method infers immune infiltration levels based on transcriptome data and is used to verify the correlation between immune genes and immune cells at the public database level as supplementary evidence for experimental detection results. Subsequently, Pearson correlation analysis was conducted between the immune infiltration scores calculated by ssGSEA and the expression levels of CDK1, B3GNT7, S100A9, and MMP9 to verify the association between immune genes and immune cell infiltration at the transcriptome level.

### 2.6 Establishment and validation of predictive models

A prognostic prediction model was constructed through binary Logistic regression analysis, with poor prognosis of thyroid cancer as the dependent variable and influencing factors as independent variables. Based on the Logistic regression model analysis results, a prognostic prediction model was constructed using formula [1/1 + exp (constant + regression coefficient (B) 1 and independent variable (X) 1 + B2X2 + B3X3 +…). The predictive effect of the model was evaluated by drawing the receiver operating characteristic (ROC) curve and using Hosmer-Lemeshow goodness of fit test.

Theoretical basis for choosing the Logistic regression model: (1) The outcome variable of this study is binary (good prognosis/poor prognosis), and no survival time variable was introduced. Therefore, the Cox proportional hazards model is not applicable. (2) Logistic regression is the standard method for constructing prediction models for binary outcomes, and it can directly estimate the effect values (OR) and confidence intervals of each factor on the outcome, with good clinical interpretability. (3) In scenarios with a limited sample size (n = 180) and a small number of predictor variables (4 genes +3 clinical variables), traditional regression models have higher stability and lower overfitting risk compared to machine learning methods.

### 2.7 Verification of public databases

To further verify the clinical significance of the immune genes screened in this study in thyroid cancer, the TCGA-THCA dataset was analyzed using the Clinical Bioinformatics Home database (https://www.aclbi.com/static/index.html#/tcga). This database integrates the transcriptome data and clinical pathological information of 512 patients with thyroid cancer from the TCGA database. The Wilcoxon rank sum test was used to compare the expression differences of CDK1, S100A9, and MMP9 in thyroid cancer tissues (n = 512) and normal thyroid tissues (n = 712). Chi-square test or Fisher’s exact test was used to analyze the distribution differences in clinical pathological characteristics (including age, gender, pT stage, pN stage, pM stage, pTNM stage, *etc.*) between the high-expression group and the low-expression group of each gene, in order to evaluate the correlation between gene expression levels and disease progression. The analysis results in this section are presented as Supplementary Materials, to provide public database-level evidence for the conclusions of our hospital’s cohort study.

### 2.8 Statistical analysis

In this study, SPSS 27.0 was used for statistical analysis. The enumeration data were represented as [cases (%)], and the sample comparison was conducted using chi square test. Measurement data that conformed to a normal distribution were represented by (mean ± standard deviation), and independent sample t-test was used for sample comparison. The influencing factors were analyzed using a binary Logistic regression model. Before modeling, the collinearity of the four immune genes was diagnosed (the variance inflation factor VIF <5). The ROC curve was drawn, and the goodness of fit of the model was evaluated by the Hosmer-Lemeshow goodness-of-fit test. The internal validation was conducted using the Bootstrap resampling method (repeated sampling 1,000 times), and the average C-index and calibration curve were calculated. The correlation was analyzed using Pearson correlation test. *P* < 0.05 was considered statistically significant.

## 3 Results

### 3.1 Comparison of clinical data

By the end of the follow-up period, among the 180 patients with thyroid cancer, 54 cases (30.0%) experienced adverse prognostic events. Among them, 26 cases (14.4%) had simple recurrence, 15 cases (8.3%) had simple distant metastasis, 9 cases (5.0%) had recurrence combined with distant metastasis, and 4 cases (2.2%) died due to thyroid cancer. The group with favorable prognosis had 126 cases (70.0%) that did not experience recurrence, metastasis or death during the follow-up period (Table 2).

**TABLE 2:** Composition of adverse events leading to poor prognosis [cases (%)].

Types of adverse prognostic events	Case	Proportion (%)
Simple recurrence	26	48.15
Simple distant metastasis	15	27.78
Recurrence combined with distant metastasis	9	16.67
Thyroid cancer-related deaths	4	7.41
Total	54	100.00

The univariate analysis showed that the proportion of patients with age>60 years, lymph node metastasis, and capsule invasion was much higher in the poor prognosis group than the good prognosis group (*P* < 0.05). The expression levels of CDK1, B3GNT7, S100A9, and MMP9 genes were significantly higher in the poor prognosis group than the good prognosis group (*P* < 0.05, Table 3).

**TABLE 3:** Comparison of clinical data [cases (%)] (‾*x* ± *s*).

Groups	Good prognosis group (n = 126)	Poor prognosis group (n = 54)	*t/χ* ^*2*^	*P*
Age (year)	​	​	​	​
≤60	60 (47.62)	12 (22.22)	10.159	0.001
>60	66 (52.38)	42 (77.78)	​	​
Gender	​	​	​	​
Male	36 (28.57)	20 (37.04)	1.264	0.261
Female	90 (71.43)	34 (62.96)	​	​
Tumor type	​	​	​	​
Follicular	30 (23.81)	12 (22.22)	3.827	0.148
Papillary	87 (69.05)	33 (61.11)	​	​
Mixed	9 (7.14)	9 (16.67)	​	​
Tumor diameter (cm)	​	​	​	​
≤3	60 (47.62)	20 (37.04)	1.714	0.190
>3	66 (52.38)	34 (62.96)	​	​
Lymph node metastasis	​	​	​	​
Yes	59 (46.83)	51 (94.44)	36.067	<0.001
No	67 (53.17)	3 (5.56)	​	​
Capsule invasion	​	​	​	​
Yes	59 (46.83)	42 (77.78)	14.705	<0.001
No	67 (53.17)	12 (2.22)	​	​
CDK1	0.68 ± 0.20	1.51 ± 0.38	19.140	<0.001
B3GNT7	1.41 ± 0.39	3.10 ± 0.76	19.679	<0.001
S100A9	1.18 ± 0.29	2.85 ± 0.70	22.679	<0.001
MMP9	0.43 ± 0.12	1.11 ± 0.32	20.748	<0.001

### 3.2 Multivariate analysis of factors affecting the prognosis of thyroid cancer patients

To prevent model estimation bias caused by multicollinearity among the predictor variables, a collinearity diagnosis was conducted on the four immune genes (CDK1, B3GNT7, S100A9, MMP9) included in the multivariate Logistic regression. The results showed that the variance inflation factors (VIF) of the CDK1, B3GNT7, S100A9, and MMP9 genes were: 2.13, 2.58, 1.96, and 2.34, respectively. All of them were less than 5, indicating that there was no severe collinearity among the variables, and the model estimation results were stable and reliable.

**FIGURE 3:**
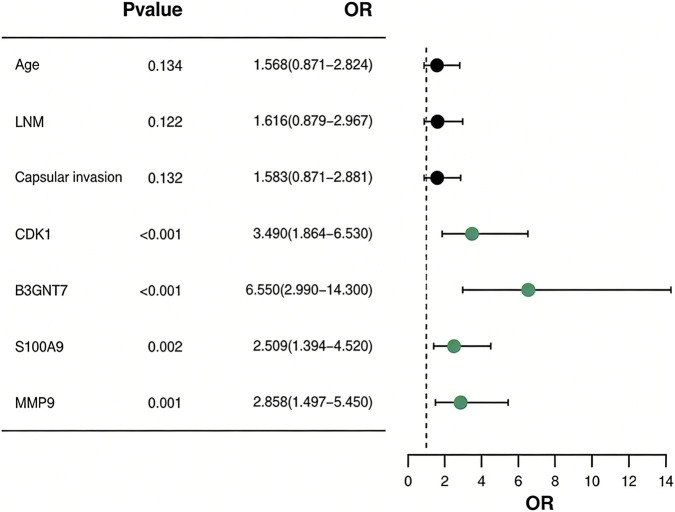
Forest plot of multivariate analysis of factors influencing the prognosis of thyroid cancer patients.

The variables with *P* < 0.05 in the univariate analysis were used as independent variables, and the prognosis of thyroid cancer patients was used as the dependent variable. The assigned values were shown in Table 4. Binary Logistic regression analysis pointed out that CDK1, B3GNT7, S100A9, and MMP9 were all independent factors affecting poor prognosis in thyroid cancer patients (*P* < 0.05, Figure 3; Table 5). Age, lymph node metastasis, and capsule invasion were not identified as independent influencing factors in the multivariate analysis (*P > 0.05*, Figure 3; Table 5).

**TABLE 4:** Assignment of independent variables.

Independent variables	​	Assignment
X1	Age	1 = ≥60 years old, 0 = <60 years old
X2	Lymph node metastasis	1 = yes, 0 = no
X3	Capsule invasion	1 = yes, 0 = no
X4	CDK1	Continuous variable
X5	B3GNT7	Continuous variable
X6	S100A9	Continuous variable
X7	MMP9	Continuous variable
Y	Prognosis	1 = poor prgonosis, 0 = good prognosis

**TABLE 5:** Multivariate analysis of factors affecting the prognosis of thyroid cancer patients.

Indicators	*B*	*SE*	*Wald*	*P*	*OR*	95% *CI*
Age	0.450	0.300	2.250	0.134	1.568	0.871–2.824
Lymph node metastasis	0.480	0.310	2.396	0.122	1.616	0.879–2.967
Capsule invasion	0.460	0.305	2.274	0.132	1.583	0.871–2.881
CDK1	1.250	0.320	15.258	<0.001	3.490	1.864–6.530
B3GNT7	1.880	0.400	22.090	<0.001	6.550	2.990–14.300
S100A9	0.920	0.300	9.406	0.002	2.509	1.394–4.520
MMP9	1.050	0.330	10.125	0.001	2.858	1.497–5.450

### 3.3 Construction and validation of prognostic model for thyroid cancer patients

**FIGURE 4:**
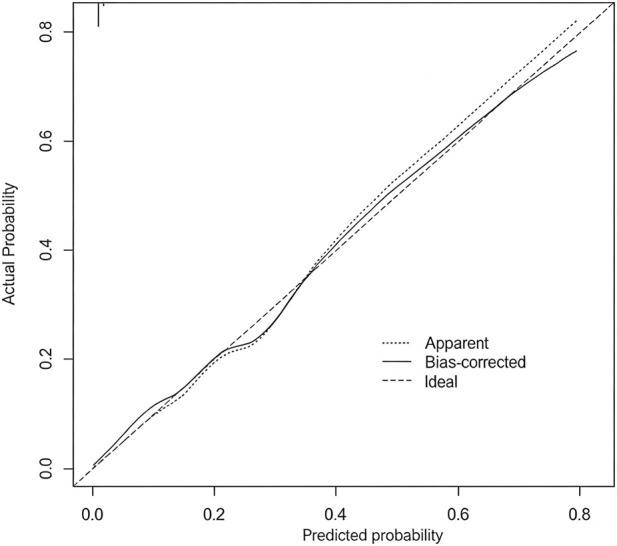
Calibration curve.

Based on the Logistic regression model, the influencing factors were screened out, and a prognostic prediction model was constructed according to formula [1/(1 + exp (4.125 + 1.250 × CDK1 + 1.880 × B3GNT7 + 0.920 × S100A9 + 1.050 × MMP9))]. To evaluate the robustness and predictive stability of the model, the Bootstrap resampling method was used for internal validation (repeated sampling 1,000 times). The validation results showed that the average C-index of the model was 0.919 (95% CI: 0.882–0.961), indicating that the model has excellent discriminatory ability, the calibration curve (Figure 4) showed that the model’s predicted probability was in good fit with the clinical observed probability, and the corresponding Hosmer-Lemeshow test result was P = 0.315, confirming that the model has ideal calibration performance and overall excellent stability.

**FIGURE 5:**
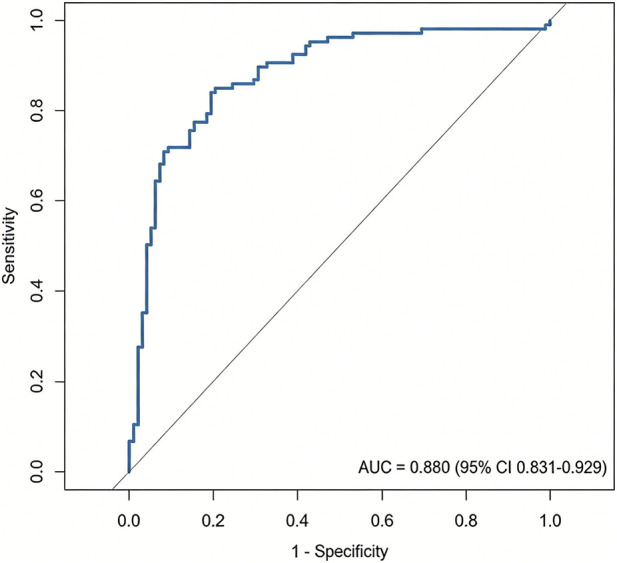
ROC curve analysis of the predictive value of the prognostic model for thyroid cancer patients.

The ROC curve was plotted for verification. The area under the ROC curve (AUC) of this prognostic prediction model was 0.880. The Hosmer-Lemeshow goodness-of-fit test was conducted, and the chi-square value was 11.246, with P = 0.257 > 0.05. This indicated that the model had good fit and had a high clinical predictive value for the risk of poor prognosis in patients with thyroid cancer (Figure 5).

### 3.4 The abundance of tumor associated immune cell infiltration in patients with different prognoses

#### 3.4.1 Immunohistochemistry combined with flow cytometry detection results

**FIGURE 6:**
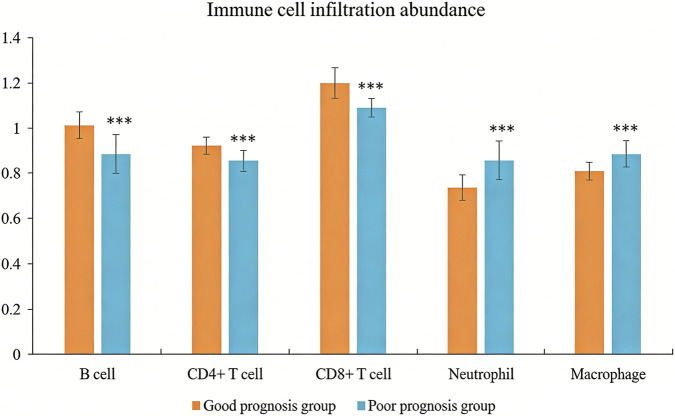
The abundance of tumor-associated immune cells infiltrating in patients with different prognoses. Compared with the group with good prognosis, *** *P* < 0.001.

The poor prognosis group had much lower infiltration abundance of B lymphocytes, CD4^+^T lymphocytes, and CD8^+^T lymphocytes, but significantly higher infiltration abundance of neutrophils and macrophages than the good prognosis group (*P* < 0.05, Figure 6).

#### 3.4.2 Results of ssGSEA bioinformatics analysis

The ssGSEA analysis of the TCGA database revealed that CDK1, B3GNT7, S100A9, and MMP9 mRNA in thyroid cancer were negatively correlated with the infiltration abundance of B lymphocytes, CD4^+^T lymphocytes, and CD8^+^T lymphocytes, and positively correlated with the infiltration abundance of neutrophils and macrophages (*P* < 0.05, Table 6).

**TABLE 6:** The correlation between immune genes and tumor associated immune cell infiltration abundance in thyroid cancer.

Indicators	CDK1	​	B3GNT7	​	S100A9	​	MMP9	​
​	*r*	*P*	*r*	*P*	*r*	*P*	*r*	*P*
B Lymphocytes	−0.240	<0.05	−0.370	<0.05	−0.485	<0.05	−0.386	<0.05
CD4+T lymphocytes	−0.274	<0.05	−0.361	<0.05	−0.289	<0.05	−0.421	<0.05
CD8+T lymphocytes	−0.145	<0.05	−0.284	<0.05	−0.360	<0.05	−0.333	<0.05
Neutrophils	0.250	<0.05	0.383	<0.05	0.281	<0.05	0.265	<0.05
Macrophages	0.336	<0.05	0.463	<0.05	0.372	<0.05	0.326	<0.05

### 3.5 Verification of the association between immune gene expression and clinical characteristics using public databases

To further verify the clinical significance of the immune genes screened in this study in thyroid cancer, the TCGA-THCA dataset was analyzed using the Clinical Bioinformatics Home database (https://www.aclbi.com/static/index.html#/tcga). The results showed that there was a significant difference in the expression levels of CDK1 and MMP9 between thyroid cancer tissues (n = 512) and normal thyroid tissues (n = 712). The expression levels of these genes in tumor tissues were significantly higher than those in normal tissues (*P* < 0.05, Supplementary Figures S1, S2). Additionally, the correlation analysis of the expression levels of S100A9 and MMP9 with the clinical and pathological characteristics of thyroid cancer patients indicated that the high expression of both was significantly associated with more advanced pN stage (lymph node metastasis), pM stage (distant metastasis), and pTNM stage (*P < 0.05*). In the high-expression groups of S100A9 and MMP9, the proportions of patients with lymph node metastasis (N1/N1a/N1b), distant metastasis (M1), and later TNM stages (III/IVA/IVC) were significantly higher (Supplementary Tables S1, S2).

## 4 Discussion

The incidence rate of thyroid cancer shows a “wave” upward trend, and the diagnosed patients are getting younger, which seriously threatens the quality of life and health of patients (Che et al., 2023). Although the postoperative prognosis of thyroid cancer is relatively good, its recurrence rate can reach 15%–20% (Park et al., 2023). Accurate identification of high-risk patients and implementation of early intervention are the key to improving prognosis. This study constructed a prognostic prediction model based on four immune-related genes, namely CDK1, B3GNT7, S100A9, and MMP9. The model had good discrimination (AUC = 0.936), satisfactory calibration (Hosmer-Lemeshow test *P* = 0.189), and an internal validation C-index of 0.921 (95% CI: 0.887–0.948), suggesting that the model has high predictive accuracy and stability. This study analyzed the association between the expression levels of these genes and the clinical and pathological characteristics of thyroid cancer using the TCGA-THCA databaseThe results showed that the expression levels of CDK1 and MMP9 in thyroid cancer tissues were significantly higher than those in normal tissues, suggesting that they might play an important role in the occurrence and development of thyroid cancer. Additionally, the high expression of S100A9 and MMP9 was significantly correlated with more advanced lymph node metastasis (pN stage), distant metastasis (pM stage), and overall TNM stage in patients. This result confirmed the potential driving role of S100A9 and MMP9 in the progression of thyroid cancer from the perspective of public databases, which was consistent with the conclusion that both are prognostic risk factors in our hospital cohort. By integrating the predictive efficacy of the hospital cohort and the molecular pathological evidence from the public database, the prognostic model based on CDK1, B3GNT7, S100A9, and MMP9 constructed in this study has a strong biological basis and clinical reference value.

The reason for selecting the four genes to construct the model in this study is that they participate in the reshaping of the immune microenvironment of thyroid cancer from different dimensions, and their combined application can comprehensively reflect the tumor’s immune escape ability. As a key kinase in the cell cycle, CDK1 high expression promotes immune checkpoint molecular membrane localization by phosphorylating PD-L1 protein, mediating tumor cell immune escape (Koyanagi et al., 2025; Cedeno-Rosario et al., 2022). B3GNT7 catalyzes β-1,3-glycosylation modification, regulates the glycosylation state of MHC-I molecules, reduces antigen presentation efficiency, and inhibits T cell recognition (Qin and Li, 2025; Wang et al., 2023). S100A9 recruits neutrophils through the CXCR4/CXCL12 axis, forming a pro-inflammatory microenvironment dominated by IL-6/IL-8 (Zhou et al., 2022; Chen et al., 2023). MMP9 degrades the extracellular matrix and releases VEGF-A, promoting the polarization of tumor-associated macrophages towards the M2 type. This not only promotes the formation of new blood vessels but also creates metastasis pathways through matrix remodeling (Fang et al., 2024; Ma et al., 2025; Guo and Jiang, 2022). The four genes respectively involve the regulation of immune checkpoints, antigen presentation, shaping of the inflammatory microenvironment, and matrix remodeling, covering the main mechanisms of tumor immune escape. Therefore, the integrated constructed model can comprehensively reflect the interaction state between the tumor and the immune system.

This study constructed a prognostic risk model for thyroid cancer based on the binary Logistic regression model. The ROC curve analysis results showed that the AUC of the combined detection of various indicators was 0.936, indicating that the established prognostic prediction model had good accuracy and high clinical application value. Theoretical basis for choosing the Logistic regression model: (1) The outcome variable of this study is binary (good prognosis/poor prognosis), and no survival time variable was introduced. Therefore, the Cox proportional hazards model is not applicable. (2) Logistic regression is the standard method for constructing prediction models for binary outcomes, and it can directly estimate the effect values (OR) and confidence intervals of each factor on the outcome, with good clinical interpretability. (3) In scenarios with a limited sample size (n = 180) and a small number of predictor variables (4 genes +3 clinical variables), traditional regression models have higher stability and lower overfitting risk compared to machine learning methods. To control multicollinearity, the collinearity diagnosis (VIF <5) was conducted for the four immune genes before modeling to ensure the stability of the model. To evaluate the robustness and predictive performance of the model, the Bootstrap resampling method was used for internal validation (repeated sampling 1,000 times), and the results showed that the average C-index of the model was 0.919 (95% CI: 0.882–0.948). The calibration curve indicated good consistency between the predicted probability and the actual observed probability (Hosmer-Lemeshow test *P* = 0.231), confirming that the model has ideal discrimination and calibration performance. Following the validation strategy of Santagata et al. (2024) in the immunotherapy prognosis model, internal validation can enhance the generalizability assessment of the model. From the perspective of clinical application value, the high sensitivity of this model (based on the OR values of various genes in Logistic regression: CDK1 is 3.490, B3GNT7 is 6.550, S100A9 is 2.509, MMP9 is 2.858) can help screen high-risk cases in the early stage of clinical practice. The regression coefficient of B3GNT7 is the highest (1.951), suggesting that its expression level has the most significant impact on prognosis and may become a core biomarker for evaluating prognosis. Clinicians can measure the expression levels of these four genes and use a formula to calculate the patient’s prognosis risk score. This allows for the early identification of high-risk individuals and the formulation of individualized follow-up and treatment strategies. For instance, patients with a high risk score may be considered for more frequent imaging follow-ups, more aggressive lymph node dissections, or exploratory immunotherapy interventions. In recent years, machine learning methods such as random survival forests, gradient boosting machines, and deep learning have been widely applied in the field of tumor prognosis prediction. However, when comparing directly between Logistic regression and machine learning, a meta-analysis published in 2025 included 28 studies on obesity risk prediction, and the results showed that the pooled AUC of machine learning methods was 0.76, while that of Logistic regression was 0.75, with no statistically significant difference between the two (Huang et al., 2025). Another systematic review on the outcome prediction of percutaneous coronary intervention also showed that there was no significant difference between machine learning and Logistic regression in predicting mortality and major adverse cardiovascular events (Jóźwiak et al., 2024). The above researches suggest that machine learning methods are not generally superior to traditional regression models in the construction of clinical prediction models. Moreover, considering the complexity of high-dimensional data, future studies can introduce dimensionality reduction methods such as Partial Least Squares Discriminant Analysis (PLS-DA) for variable selection and model optimization. This method has been proven effective in similar biomarker studies (Fordellone et al., 2020).

This study also found that the poor prognosis group had significantly lower infiltration abundance of B lymphocytes, CD4^+^T lymphocytes, and CD8^+^T lymphocytes, and much higher infiltration abundance of neutrophils and macrophages than the good prognosis group. This result suggested that immune genes might play an important role in regulating the immune microenvironment of thyroid cancer, and might participate in tumor occurrence, development, and immune escape by affecting the infiltration and function of immune cells. High expression of CDK1 promotes membrane localization and stability by phosphorylating PD-L1 serine at position 198. In this study, CDK1 was negatively correlated with CD8^+^T cell infiltration (r = −0.145, *P* < 0.05), confirming that high expression of PD-L1 could bind to PD-1 on the surface of CD8^+^T cells and inhibit their killing function. In addition, CDK1 can phosphorylate transcription factor NF-κB, downregulate the expression of chemokine CXCL10, and reduce T cell recruitment (García-Gutiérrez et al., 2019). *In vitro* experiments (Zhuang et al., 2020) have shown that B3GNT7 knockout can restore the glycosylation pattern of MHC-I molecules in lung adenocarcinoma cells and enhance CD8^+^T cell recognition. This is consistent with the mechanism of the negative correlation (*r* = −0.370, *P* < 0.05) between B3GNT7 and B lymphocyte infiltration in our present study. As antigen-presenting cells, the reduced infiltration of B lymphocytes may further weaken tumor immune surveillance. In this study, S100A9 was positively correlated with neutrophil infiltration (r = 0.281, *P* < 0.05). The immune infiltration characteristics provide an explanation for the biological basis of the model. The high expression of four genes creates an immunosuppressive microenvironment, manifested by reduced lymphocyte infiltration and increased myeloid cell infiltration, which enables tumor cells to evade the host immune surveillance and ultimately leads to an increased risk of recurrence and metastasis. The results of immune infiltration analysis and the predictive performance of the model are mutually confirmed, indicating that the model not only has statistical predictive ability, but also has clear immunological mechanism support (Zha et al., 2019; Rouas et al., 2019).

In general, CDK1, B3GNT7, S100A9, and MMP9 were risk factors for poor prognosis in thyroid cancer. Immune genes were associated with the abundance of immune cell infiltration. The prognosis prediction model constructed based on various indicators can quantify the risk of poor prognosis in patients and provide objective basis for early screening of high-risk cases in clinical practice. There was a significant correlation between the expression of immune genes in thyroid cancer and the immune infiltration characteristics of the tumor microenvironment. This study revealed the potential mechanism by which immune genes participated in the progression of thyroid cancer *via* regulating the dynamic infiltration of immune cells. We provided a new research direction for the development of personalized immunotherapy strategies based on immune gene expression profiles.

This study also had limitations. Firstly, the sample source was single and the included research subjects were all from the patient population of our hospital, which might limit the generalizability of the research results. Secondly, this study was a single center retrospective study, which might have selection bias. It is recommended that a multi-center prospective research model should be adopted in the following study to further validate the clinical applicability of the predictive model by expanding the sample size and geographic representation. Although this study validated the differential expression of CDK1, MMP9, and S100A9 and their association with clinical pathological features through the TCGA-THCA database, the validation content of this database mainly focuses on the correlation between expression levels and clinical pathological features, and has not been included in other public databases such as GEO for external validation. Moreover, the survival analysis of the four genes in the TCGA database did not reach statistical significance. Further studies need to include external data from multiple centers to comprehensively evaluate the robustness and universality of the model.

## Data Availability

The original contributions presented in the study are included in the article/Supplementary Material, further inquiries can be directed to the corresponding author.

## Supplementary material

SUPPLEMENTARY FIGURE S1 Differential expression of CDK1 between thyroid cancer tissue and normal tissue.

SUPPLEMENTARY FIGURE S2 Differential expression of MMP9 between thyroid cancer tissue and normal tissue.

## References

[B1] Alonso-Gordoa T. Jimenez-Fonseca P. Martinez-Trufero J. Navarro M. Porras I. Rubió-Casadevall J. (2024). SEOM-GETNE-TTCC clinical guideline thyroid cancer. Clin. Transl. Oncol. 26 (11), 2902–2916. 10.1007/s12094-024-03736-6 39325263 PMC11467120

[B2] Boucai L. Zafereo M. Cabanillas M. E. (2024). Thyroid cancer: a review. JAMA 331 (5), 425–435. 10.1001/jama.2023.26348 38319329

[B3] Cedeno-Rosario L. Honda D. Sunderland A. M. Lewandowski M. D. Taylor W. R. Chadee D. N. (2022). Phosphorylation of mixed lineage kinase MLK3 by cyclin-dependent kinases CDK1 and CDK2 controls ovarian cancer cell division. J. Biol. Chem. 298 (8), 102263. 10.1016/j.jbc.2022.102263 35843311 PMC9399292

[B4] Chen D. W. Lang B. H. H. McLeod D. S. A. Newbold K. Haymart M. R. (2023). Thyroid cancer. Lancet 401 (10387), 1531–1544. 10.1016/S0140-6736(23)00020-X 37023783

[B5] Chen Y. Ouyang Y. Li Z. Wang X. Ma J. (2023). S100A8 and S100A9 in cancer. Biochim. Biophys. Acta Rev. Cancer 1878 (3), 188891. 10.1016/j.bbcan.2023.188891 37001615

[B6] Fang S. Du S. Luo X. Qing X. Wang L. Ban Y. (2024). The role of the S100A8/S100A9 in gastric tumor progression. Sci. Rep. 14 (1), 23574. 10.1038/s41598-024-74695-9 39384957 PMC11464527

[B7] Fordellone M. Bellincontro A. Mencarelli F. (2020). Partial least squares discriminant analysis: a dimensionality reduction method to classify hyperspectral data. Statistica Appl. - Italian J. Appl. Statistics 29 (10), 181–200. 10.48550/arXiv.1806.09347

[B8] García-Gutiérrez L. Bretones G. Molina E. Arechaga I. Symonds C. Acosta J. C. (2019). Myc stimulates cell cycle progression through the activation of Cdk1 and phosphorylation of p27. Sci. Rep. 9 (1), 18693. 10.1038/s41598-019-54917-1 31822694 PMC6904551

[B9] Guo Z. Y. Jiang L. P. (2022). Matrix metalloproteinase 12 (MMP12) as an adverse prognostic biomarker of vascular invasion in hepatic cell carcinoma. Eur. Rev. Med. Pharmacol. Sci. 26 (7), 2238–2249. 10.26355/eurrev_202204_28454 35442508

[B10] Huang Y. Bazzazzadehgan S. Li J. Arabshomali A. Li M. Bhattacharya K. (2025). Comparison of machine learning methods *versus* traditional cox regression for survival prediction in cancer using real-world data: a systematic literature review and meta-analysis. BMC Med. Res. Methodol. 25 (1), 243. 10.1186/s12874-025-02694-z 41152747 PMC12570641

[B11] Ito Y. Arai K. Nozawa R. Yoshida H. Hirokawa M. Fukushima M. (2009). S100A8 and S100A9 expression is a crucial factor for dedifferentiation in thyroid carcinoma. Anticancer Res. 29 (10), 4157–4161. Available online at: https://ar.iiarjournals.org/lookup/pmidlookup?view=long&pmid=19846966 19846966

[B12] Jóźwiak K. Nguyen V. H. Sollfrank L. Linn S. C. Hauptmann M. (2024). Cox proportional hazards regression in small studies of predictive biomarkers. Sci. Rep. 14 (1), 14232. 10.1038/s41598-024-64573-9 38902269 PMC11190253

[B13] Koyanagi N. Hengphasatporn K. Kato A. Nobe M. Takeshima K. Maruzuru Y. (2025). Regulatory mimicry of cyclin-dependent kinases by a conserved herpesvirus protein kinase. Proc. Natl. Acad. Sci. U. S. A. 122 (16), e2500264122. 10.1073/pnas.2500264122 40238458 PMC12037052

[B14] Ma Z. Zhang J. Li Z. Zhu Y. Han X. Lei L. (2025). Interleukin-1β inhibits ovarian cancer cell proliferation and metastasis through the MAPK/MMP12 pathway. Int. J. Mol. Sci. 26 (7), 3287. 10.3390/ijms26073287 40244135 PMC11989259

[B15] Mallardo D. Fordellone M. White A. Ottaviano M. Sparano F. Bailey M. (2023). CD39 and LDHA affects the prognostic role of NLR in metastatic melanoma patients treated with immunotherapy. J. Transl. Med. 21 (1), 610. 10.1186/s12967-023-04419-6 37684649 PMC10492378

[B16] Mallardo D. Fordellone M. White A. Vowinckel J. Bailey M. Sparano F. (2024). A combined proteomic and transcriptomic signature is predictive of response to anti-PD-1 treatment: a retrospective study in metastatic melanoma patients. Int. J. Mol. Sci. 25 (17), 9345. 10.3390/ijms25179345 39273294 PMC11395026

[B17] Nagae M. Kizuka Y. Mihara E. Kitago Y. Hanashima S. Ito Y. (2018). Structure and mechanism of cancer-associated N-acetylglucosaminyltransferase-V. Nat. Commun. 9 (1), 3380. 10.1038/s41467-018-05931-w 30140003 PMC6107550

[B18] Park J. Kang I. K. Bae J. S. Kim J. S. Kim K. (2023). Clinical significance of the lymph node ratio of the second operation to predict re-recurrence in thyroid carcinoma. Cancers (Basel) 15 (3), 624. 10.3390/cancers15030624 36765580 PMC9913116

[B19] Pizzato M. Li M. Vignat J. Laversanne M. Singh D. La Vecchia C. (2022). The epidemiological landscape of thyroid cancer worldwide: GLOBOCAN estimates for incidence and mortality rates in 2020. Lancet Diabetes Endocrinol. 10 (4), 264–272. 10.1016/S2213-8587(22)00035-3 35271818

[B20] Qin J. Li Z. (2025). Identification of CDK1 as a biomarker for the treatment of liver fibrosis and hepatocellular carcinoma through bioinformatics analysis. Int. J. Mol. Sci. 26 (8), 3816. 10.3390/ijms26083816 40332418 PMC12028024

[B21] Rouas R. Merimi M. Najar M. El Zein N. Fayyad-Kazan M. Berehab M. (2019). Human CD8^+^ CD25 + CD127 low regulatory T cells: microRNA signature and impact on TGF-β and IL-10 expression. J. Cell Physiol. 234 (10), 17459–17472. 10.1002/jcp.28367 30805923

[B22] Santagata S. Trotta A. M. d'Alterio C. Napolitano M. Rea G. Di Napoli M. (2024). KIR2DL2/DL3+ NKs and Helios+ Tregs in peripheral blood predict nivolumab response in patients with metastatic renal cell cancer. Clin. Cancer Res. 30 (20), 4755–4767. 10.1158/1078-0432.CCR-24-0729 39167621 PMC11474171

[B23] Toraih E. A. Hussein M. H. Al Ageeli E. Ellaban M. Kattan S. W. Moroz K. (2023). Matrix metalloproteinase 9/microRNA-145 ratio: bridging genomic and immunological variabilities in thyroid cancer. Biomedicines 11 (11), 2953. 10.3390/biomedicines11112953 38001954 PMC10669161

[B24] Wang X. Wang Y. Chen X. He Y. Zhou X. Jiao S. (2023). Identification of glycogene-based prognostic signature and validation of B3GNT7 as a potential biomarker and therapeutic target in breast cancer. J. Cancer Res. Clin. Oncol. 149 (19), 16957–16969. 10.1007/s00432-023-05345-2 37740763 PMC11797399

[B25] Xiang C. Sun W. H. Ke Y. Yu X. Wang Y. (2022). CDCA8 contributes to the development and progression of thyroid cancer through regulating CDK1. J. Cancer 13 (7), 2322–2335. 10.7150/jca.64747 35517403 PMC9066215

[B26] Xu Q. Yan X. Han Z. Jin X. Jin Y. Sun H. (2022). Immune cell infiltration and relevant gene signatures in the tumor microenvironment that significantly associates with the prognosis of patients with breast cancer. Front. Mol. Biosci. 9, 823911. 10.3389/fmolb.2022.823911 35281270 PMC8905140

[B27] Zha H. Li X. Sun H. Duan L. Yuan S. Li H. (2019). S100A9 promotes the proliferation and migration of cervical cancer cells by inducing epithelial-mesenchymal transition and activating the Wnt/β-catenin pathway. Int. J. Oncol. 55 (1), 35–44. 10.3892/ijo.2019.4793 31059008 PMC6561615

[B28] Zhou H. Zhao J. Yang X. Liu J. Huang W. (2022). Study on the expression of β-1,3-N-acetylglucosaminyltransferase 3 in gastric cancer and the mechanism promoting gastric cancer progression based on the extraction method of nanomagnetic beads. J. Biomed. Nanotechnol. 18 (3), 677–692. 10.1166/jbn.2022.3296 35715910

[B29] Zhuang H. Zhou Z. Zhang Z. Chen X. Ma Z. Huang S. (2020). B3GNT3 overexpression promotes tumor progression and inhibits infiltration of CD8+ T cells in pancreatic cancer. Aging (Albany NY) 13 (2), 2310–2329. 10.18632/aging.202255 33316775 PMC7880340

