# Supplementary figures and images for: Charting the immune terrain: a novel risk model for thyroid cancer prognosis
Source: Front Genet. 2026 Apr 23;17:1752017. doi: 10.3389/fgene.2026.1752017 (PMC13148796; doi:10.3389/fgene.2026.1752017)

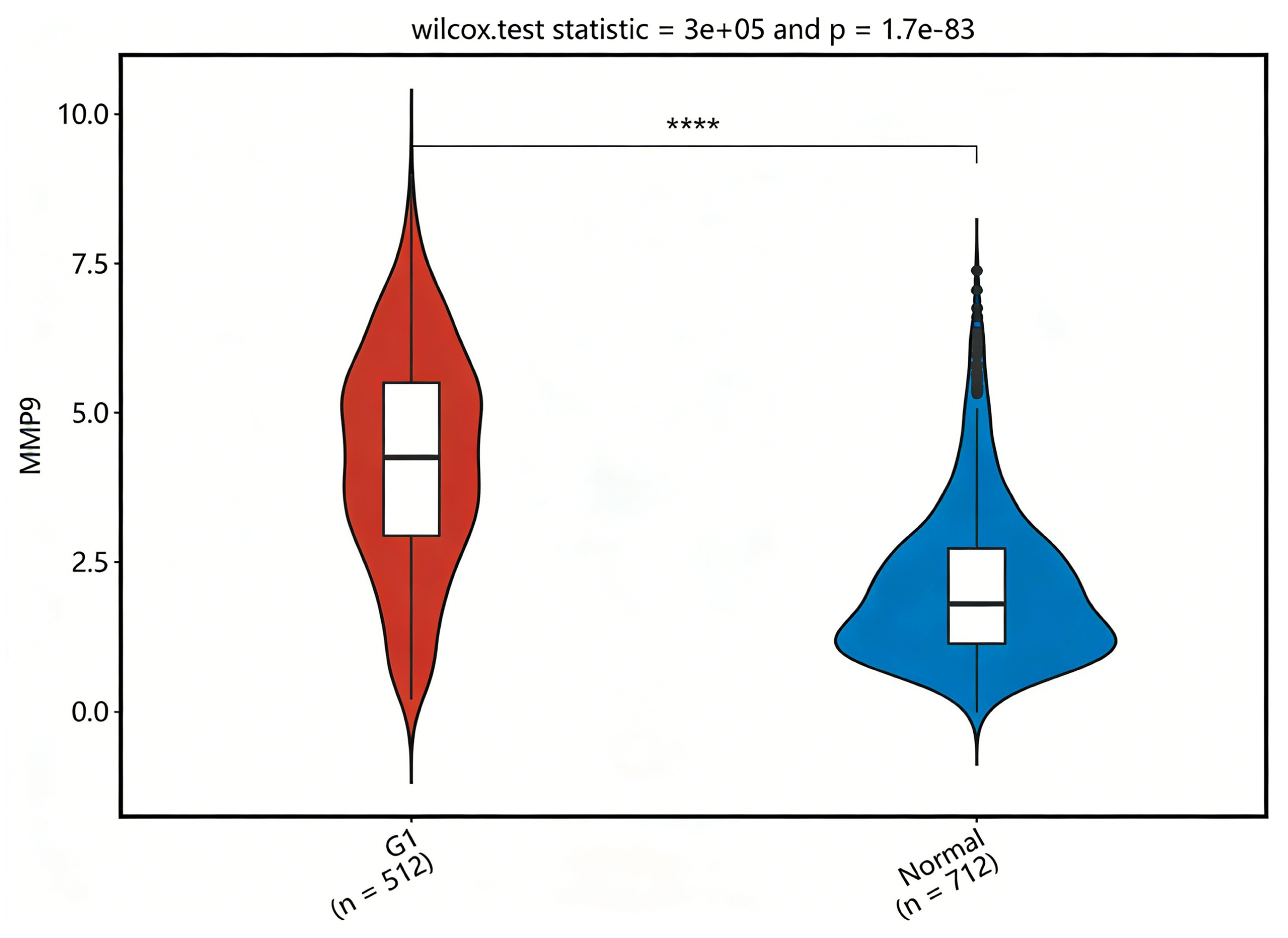

Supplement: Supplementary file 1 [file Image2.jpg]

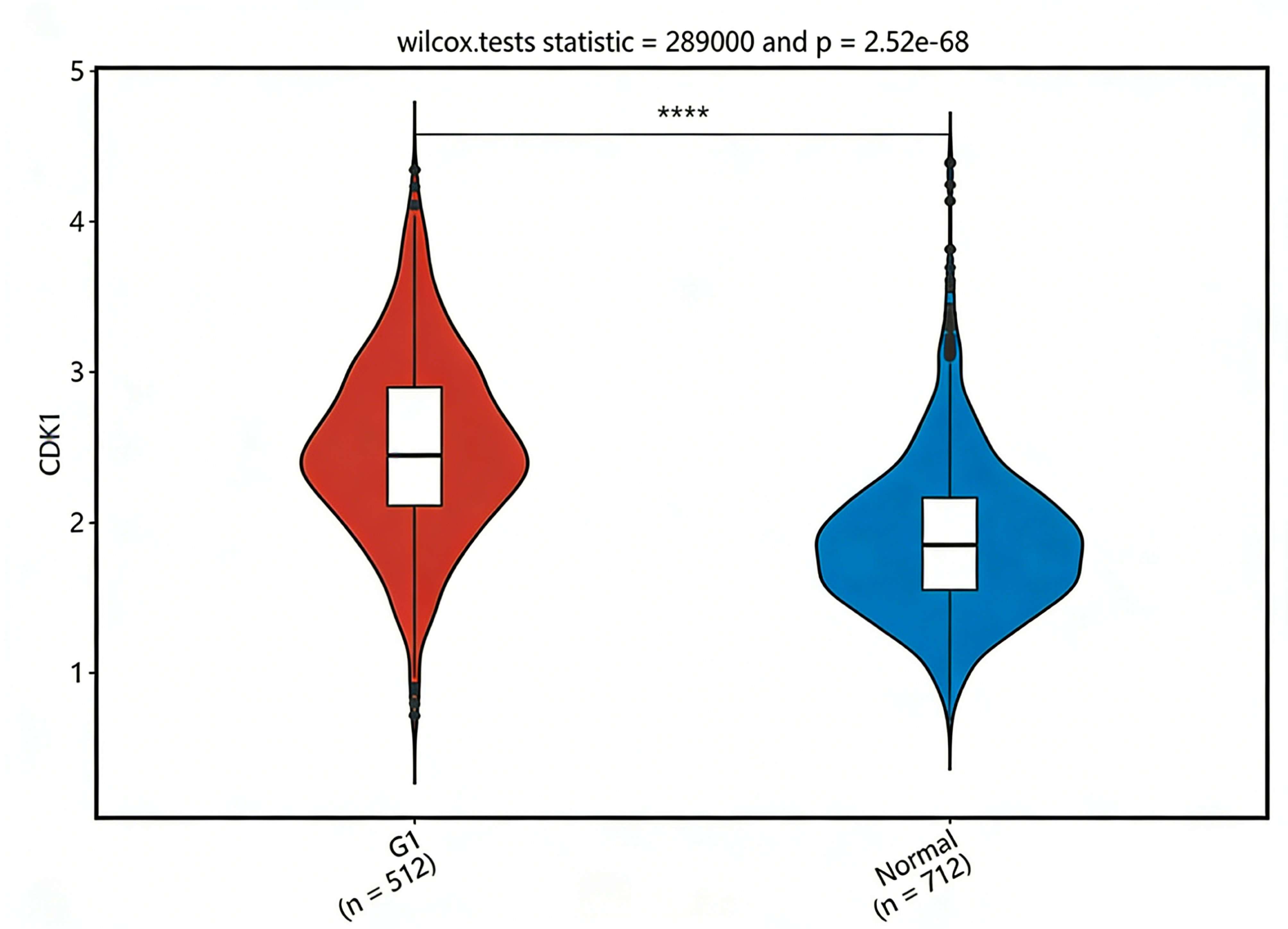

Supplement: Supplementary file 3 [file Image1.jpg]
