# Supplementary material for: Charting the immune terrain: a novel risk model for thyroid cancer prognosis
Source: Front Genet. 2026 Apr 23;17:1752017. doi: 10.3389/fgene.2026.1752017 (PMC13148796; doi:10.3389/fgene.2026.1752017)
Supplement: Supplementary file 2 [file Table1.doc]

**Supplementary Table 1 Association between high and low expression of S100A9 and clinical pathological characteristics of thyroid cancer patients (from TCGA database)**

|  | Charar | S100A9 High expression  N=256 | S100A9  Low expression  N=256 | P_value |
| --- | --- | --- | --- | --- |
| Status | Alive | 247 | 249 |  |
|  | Dead | 9 | 7 | 0.799 |
| Age | Mean (SD) | 46.6 (16.2) | 47.8 (15.4) |  |
|  | Median [MIN, MAX] | 46 [15,89] | 47 [15,85] | 0.404 |
| Gender | FEMALE | 185 | 188 |  |
|  | MALE | 71 | 68 | 0.842 |
| Race | AMERICAN INDIAN | 1 |  |  |
|  | ASIAN | 21 | 30 |  |
|  | BLACK | 16 | 11 |  |
|  | WHITE | 187 | 151 | 0.142 |
| pT_stage | T1 | 16 | 28 |  |
|  | T1a | 11 | 8 |  |
|  | T1b | 43 | 37 |  |
|  | T2 | 84 | 85 |  |
|  | T3 | 89 | 86 |  |
|  | T4 | 5 | 4 |  |
|  | T4a | 7 | 7 |  |
|  | TX | 1 | 1 | 0.737 |
| pN_stage | N0 | 108 | 121 |  |
|  | N1 | 35 | 24 |  |
|  | N1a | 50 | 42 |  |
|  | N1b | 48 | 34 |  |
|  | NX | 15 | 35 | 0.008 |
| pM_stage | M0 | 157 | 129 |  |
|  | M1 | 2 | 7 |  |
|  | MX | 97 | 119 | 0.021 |
| pTNM_stage | I | 148 | 140 |  |
|  | II | 14 | 38 |  |
|  | III | 64 | 49 |  |
|  | IV | 1 | 1 |  |
|  | IVA | 28 | 21 |  |
|  | IVC | 1 | 5 | 0.005 |
| new_tumor_  event_type | Metastasis | 6 | 8 |  |
|  | Primary | 4 | 2 |  |
|  | Recurrence | 13 | 13 | 0.621 |
