# Supplementary material for: Charting the immune terrain: a novel risk model for thyroid cancer prognosis
Source: Front Genet. 2026 Apr 23;17:1752017. doi: 10.3389/fgene.2026.1752017 (PMC13148796; doi:10.3389/fgene.2026.1752017)
Supplement: Supplementary file 4 [file Table2.doc]

**Supplementary Table 2 Association between high and low expression of MMP9 and the clinical pathological characteristics of thyroid cancer patients (TCGA database)**

|  | Charar | MMP9  High expression | MMP9  Low expression | P_value |
| --- | --- | --- | --- | --- |
| Status | Alive | 247 | 249 |  |
|  | Dead | 9 | 7 | 0.799 |
| Age | Mean (SD) | 47.4 (15.8) | 47 (15.9) |  |
|  | Median [MIN, MAX] | 46 [15,89] | 46.5 [15,85] | 0.766 |
| Gender | FEMALE | 187 | 186 |  |
|  | MALE | 69 | 70 | 1 |
| Race | AMERICAN INDIAN | 1 |  |  |
|  | ASIAN | 29 | 22 |  |
|  | BLACK | 9 | 18 |  |
|  | WHITE | 184 | 154 | 0.093 |
| pT_stage | T1 | 22 | 22 |  |
|  | T1a | 10 | 9 |  |
|  | T1b | 43 | 37 |  |
|  | T2 | 69 | 100 |  |
|  | T3 | 96 | 79 |  |
|  | T4 | 6 | 3 |  |
|  | T4a | 9 | 5 |  |
|  | TX | 1 | 1 | 0.19 |
| pN_stage | N0 | 103 | 126 |  |
|  | N1 | 33 | 26 |  |
|  | N1a | 56 | 36 |  |
|  | N1b | 48 | 34 |  |
|  | NX | 16 | 34 | 0.003 |
| pM_stage | M0 | 156 | 130 |  |
|  | M1 | 5 | 4 |  |
|  | MX | 95 | 121 | 0.061 |
| pTNM_stage | I | 144 | 144 |  |
|  | II | 15 | 37 |  |
|  | III | 62 | 51 |  |
|  | IV | 1 | 1 |  |
|  | IVA | 30 | 19 |  |
|  | IVC | 4 | 2 | 0.019 |
| new_tumor_event_type | Metastasis | 11 | 3 |  |
|  | Primary | 3 | 3 |  |
|  | Recurrence | 16 | 10 | 0.393 |
